# Longitudinal Monitoring of Donor-Derived Cell-Free DNA Supports Risk Stratification in Kidney Transplant Recipients With Allograft Dysfunction

**DOI:** 10.3389/ti.2026.15929

**Published:** 2026-03-12

**Authors:** Iris Schröter, Lisa Loi, Marvin Reineke, Markus Rudek, Christian Nusshag, Florian Kälble, Claudius Speer, Martin Zeier, Thuong Hien Tran, Christian Morath, Louise Benning

**Affiliations:** 1 Department of Nephrology, University Hospital Heidelberg, Heidelberg, Germany; 2 Department of Transplantation Immunology, University Hospital Heidelberg, Heidelberg, Germany; 3 Department of Nephrology and Hypertension, Klinikum Nuremberg, Paracelsus Medical University, Nuremberg, Germany

**Keywords:** dd-cfDNA, donor-derived cell-free DNA, graft failure, kidney transplantation, rejection

## Abstract

The prognostic value of donor-derived cell-free DNA (dd-cfDNA) for long-term kidney allograft outcomes after indication biopsy remains incompletely defined. In this prospective single-center cohort, 106 kidney transplant recipients with 108 indication biopsies were assessed for dd-cfDNA at biopsy and at 7, 30, and 90 days thereafter. dd-cfDNA was analyzed as a continuous, threshold-based, and longitudinal time-dependent variable. Clinical endpoints included ≥30% eGFR decline within 2 years, indication for re-biopsy, and graft failure. Persistent elevation of dd-cfDNA (≥0.5% at 90 days) occurred in 7.4% of patients, with 50% requiring re-biopsy and 37.5% developing graft failure. A single measurement ≥1.0% significantly predicted ≥30% eGFR decline (HR 2.28; 95% CI 1.03–5.05), whereas levels ≥0.5% were less discriminative. In multivariable time-dependent Cox models adjusted for age, sex, time from transplantation to biopsy, baseline eGFR, baseline proteinuria, and Banff domain scores, longitudinal dd-cfDNA remained independently associated with ≥30% eGFR decline (HR 1.68; 95% CI 1.12–2.51), re-biopsy (HR 1.88; 95% CI 1.38–2.55), and graft failure (HR 3.42; 95% CI 2.00–5.86). In conclusion, dd-cfDNA levels, particularly when assessed longitudinally, are associated with adverse allograft outcomes after indication biopsy and may provide relevant prognostic information beyond a single measurement.

## Graphical Abstract

**Figure:**
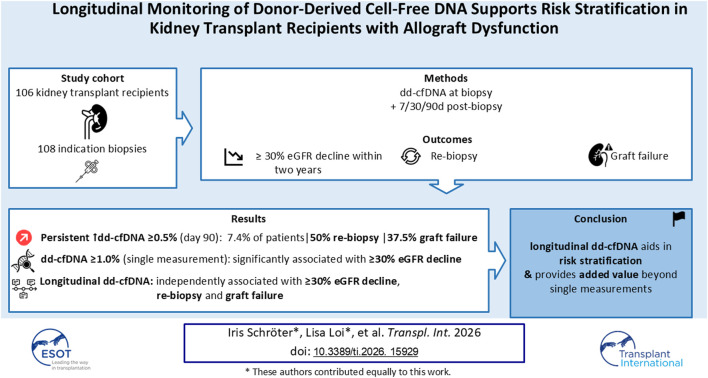

## Introduction

Donor-derived cell-free DNA (dd-cfDNA) has emerged as a promising non-invasive biomarker for monitoring kidney allograft health, and has recently been shown to improve clinical decision-making beyond the standard of care in kidney transplantation [1–4]. Elevated levels of dd-cfDNA in the recipient’s bloodstream, released during graft cell death, reflect ongoing graft injury, for example in cases of rejection, and often precede clinical or histological changes [5, 6]. Several studies have demonstrated the utility of dd-cfDNA in detecting acute rejection, particularly antibody-mediated rejection (AMR) [2–4, 7–11] with increasing dd-cfDNA levels reflecting the severity of microcirculation inflammation [1, 3, 5, 12]. Additionally, decreasing dd-cfDNA levels have been shown to indicate responses to rejection treatment and have been incorporated into recent clinical trials targeting AMR [13–17]. Further, there is emerging evidence that dd-cfDNA may also have prognostic value, for example in relation to the development of *de novo* donor-specific antibodies (DSA) and a subsequent decline in estimated glomerular filtration rate (eGFR) [18, 19].

However, data regarding the utility of particularly sequential dd-cfDNA measurements for longer-term outcomes such as graft failure, progressive allograft dysfunction indicating re-biopsy, or long-term eGFR decline remain limited. It is unclear whether fixed absolute thresholds are sufficient or whether the rate and magnitude of change over time (kinetics) provide greater prognostic value, and how dd-cfDNA testing should be optimally integrated into clinical decision-making.

To address these gaps, we analyzed both single time-point and longitudinal dd-cfDNA measurements in kidney transplant recipients with indication biopsies, evaluating dd-cfDNA as a continuous variable and through various threshold-based and longitudinal models to assess associations with key clinical outcomes.

## Materials and Methods

### Study Design

This prospective single-center study included 106 kidney transplant recipients from the Department of Nephrology at Heidelberg University Hospital who underwent 108 clinically indicated allograft biopsies between November 2020 and December 2022. Biopsies were examined by two board-examined pathologists and reported using the BANFF 2018 reference guide [20]. Histopathology was not re-scored using later Banff updates, as clinical management during the study period was based on Banff 2018.

dd-cfDNA was measured at the time of biopsy (T_0_) and at follow-up visits 7- (T_1_), 30- (T_2_), and 90-day (T_3_) post-biopsy. Throughout the study period, treating physicians were blinded to dd-cfDNA testing results, as dd-cfDNA was measured for research purposes only and was not implemented in routine clinical care at our center. To quantify dd-cfDNA, plasma was isolated by sequential centrifugation and either stored at −80 °C or processed immediately for cfDNA extraction. dd-cfDNA was quantified using the AlloSeq cfDNA assay (CareDx), a multiplex PCR-based assay targeting 202 single nucleotide polymorphisms (SNPs). Sequence data was analyzed using the CareDx AlloSeq cfDNA software and all procedures were performed as described previously [4].

Clinical parameters, including serum creatinine, eGFR, and proteinuria, were assessed at the same initial time points and additionally at 180 days, 1 year, 2 years, and 3 years post-biopsy as part of an ongoing longitudinal follow-up. Detailed descriptions of the study setting as well as the results focusing on the initial follow-up period up to day 180, and correlations with histopathology have been published previously [4].

Expanding on our initial short-term findings, this analysis explores longer-term dd-cfDNA patterns and their association with clinical outcomes beyond the early post-biopsy period. To explore these associations, patients were categorized into three groups based on dd-cfDNA levels: <0.5%, ≥0.5% to <1.0%, and ≥1.0%. These thresholds were applied to ensure comparability with previously published studies, in which these thresholds were used to evaluate dd-cfDNA as a prognostic marker and risk stratification tool in kidney transplant patients [18, 21, 22]. We then analyzed whether dd-cfDNA levels at biopsy, as well as their trajectories over time, could predict a ≥30% decline in eGFR slope 2 years post-biopsy, progressive allograft dysfunction requiring repeat biopsy, or the event of graft failure.

The study was approved by the ethics committee of the University of Heidelberg and conducted in accordance with the Declaration of Helsinki. Written informed consent was obtained from all study participants. The study is registered in the German Clinical Trials Register (DRKS00023604).

### Statistical Analysis

Descriptive statistics were used to summarize baseline characteristics of the study population. Continuous variables were reported as mean ± standard deviation (SD) or median with interquartile range (IQR), depending on their distribution. Categorical variables were presented as counts and percentages. Group comparisons were performed using t-tests or Mann–Whitney *U* tests for continuous variables and chi-square or Fisher’s exact tests for categorical variables, as appropriate.

Time-to-event outcomes were analyzed using Kaplan-Meier survival curves, with differences between groups assessed using the log-rank test. Time was defined as the interval from the initial biopsy to either the last clinical follow-up or the occurrence of the clinical endpoint. Missing dd-cfDNA values were not imputed, and patients contributed person-time only for intervals with an observed dd-cfDNA measurement. To assess potential bias from incomplete follow-up, baseline characteristics were compared between patients with complete versus incomplete dd-cfDNA sampling.

Three clinical endpoints were analyzed using Cox proportional hazards regression. Time was defined as the interval from the index biopsy to the occurrence of the respective endpoint or last available follow-up. Three clinical endpoints were examined: (A) a ≥30% decline in eGFR within 2 years after biopsy, (B) clinical indication for re-biopsy, and (C) graft failure.

To assess the association between dd-cfDNA and these outcomes, dd-cfDNA was evaluated using several complementary approaches:

- dd-cfDNA was analyzed as a continuous variable measured at the initial timepoint (T_0_).
- Threshold-based stratifications were applied using cutoffs of ≥0.5% and ≥1.0% at biopsy, reflecting commonly used thresholds where levels ≥0.5% suggest likely graft injury and levels ≥1.0% indicate a high risk of rejection [7, 18, 22, 23].
- To evaluate the impact of sustained elevation, “persistently high dd-cfDNA” was defined as values ≥0.5% at all measured timepoints (at biopsy as well as 7-, 30-, and 90-day post-biopsy).
- The increase in mean dd-cfDNA from the time of biopsy to 90 days post-biopsy was calculated and dichotomized at >0.3%. This cutoff was chosen to allow discrimination in a cohort with dd-cfDNA values predominantly <0.5% and is supported by prior evidence that rises >61% exceed the reference change value (RCV) for dd-cfDNA [24].
- Longitudinal dd-cfDNA trajectories were analyzed using time-dependent Cox regression models. For this purpose, dd-cfDNA measurements obtained at biopsy and during follow-up (7, 30, and 90 days) were reformatted into a start-stop data structure, with each interval assigned the most recent dd-cfDNA value. This approach allows for dynamic modeling of evolving dd-cfDNA levels in relation to subsequent clinical events. Univariable Cox models were initially fitted for each endpoint to explore associations between dd-cfDNA, clinical variables, laboratory measures, and histopathological findings. Multivariable models were then constructed using a prespecified, clinically motivated adjustment strategy. All primary multivariable models included recipient age and sex, time from transplantation to biopsy, baseline graft function, and baseline proteinuria, as these variables are plausibly associated with both dd-cfDNA levels and the studied endpoints. Histopathology was incorporated using aggregated Banff-based domain scores to reduce collinearity and avoid overfitting given the limited number of outcome events. TCMR-related activity/tubulointerstitial inflammation was summarized as the combined score of interstitial inflammation, tubulitis, and intimal arteritis (t + i + v); AMR-related activity/microvascular inflammation (MVI) was defined as the sum of glomerulitis, peritubular capillaritis und C4d positivity (g + ptc + C4d); and chronic injury burden was captured using a composite of Banff lesions indicating chronicity (ci + ct + cv + cg). To assess the robustness of the findings, prespecified sensitivity analyses were performed, including models excluding all histological variables and models adjusting only for chronic injury burden. Proportional hazards assumptions were assessed using Schoenfeld residuals for all multivariable models. Model discrimination was evaluated using Harrell’s concordance index (C-index). Results are reported as hazard ratios (HRs) with 95% confidence intervals (CIs; see Supplementary Material).

All statistical analyses were conducted using R Statistical Software (Version 2024.12.0 + 467).

## Results

### Baseline Characteristics and dd-cfDNA Trajectories in Relation to Histopathology

A total of 106 kidney transplant recipients undergoing 108 indication biopsies with concurrent dd-cfDNA measurement were included. The mean age at biopsy was 49 ± 2 years, and 35/106 (33%) were female. Median time from transplantation to biopsy was 963 days (IQR 97–2853). The median clinical follow-up was 832 days (IQR 486–1,074).

Table 1 summarizes the clinical and demographic characteristics of the entire cohort as well as subgroups stratified by dd-cfDNA thresholds (<0.5% and ≥0.5%) at the time of indication biopsy. Compared to patients with dd-cfDNA <0.5%, those with levels ≥0.5% exhibited significantly lower eGFR (*P* = 0.004), a higher prevalence of preformed antibodies (*P* = 0.031), and an increased incidence of acute rejection (*P* = 0.040), particularly AMR (*P* = 0.043). No other baseline characteristics differed significantly between the two groups. Baseline characteristics between patients with complete versus incomplete dd-cfDNA follow-up were compared to assess potential bias but showed no significant differences (data not shown).

**TABLE 1:** Patient characteristics stratified by dd-cfDNA thresholds at time of biopsy (T_0_).

Variable	Total cohort N = 106	dd-cfDNA <0.5 N = 70	dd-cfDNA ≥0.5 N = 36	*P*-value	Available (N)
Demographics at time of biopsy (T_0_)					​
Age	49.2 ± 14.5	50.5 ± 14.8	46.5 ± 13.8	0.181	106
Male sex	71 (67.0)	50 (71.4)	21 (58.3)	0.254	106
Body mass index (kg/m^2^)	26.0 ± 4.7	26.5 ± 5.0	25.0 ± 4.0	0.138	106
Prior Tx	16 (15.4)	7 (10.1)	9 (25.0)	0.061	104
Donor data					
Donor age	55.1 ± 13.4	55.3 ± 13.3	54.7 ± 13.8	0.842	96
Male donor	44 (44.0)	29 (43.9)	15 (44.1)	1.000	100
AB0i Tx	3 (2.9)	3 (4.3)	0 (0.0)	0.549	104
Deceased donation	68 (64.2)	44 (62.9)	24 (66.7)	0.089	106
Laboratory					
Baseline serum creatinine (mg/dL)	2.0 ± 0.8	1.9 ± 0.7 (62)	2.2 ± 1.0 (28)	0.091	90
T_0_ serum creatinine (mg/dL)	3.0 ± 2.3	2.7 ± 1.7	3.7 ± 3.1	0.079	106
T_0_ proteinuria (g/molCr)	141.8 ± 219.7	112.7 ± 158.1	209.3 ± 314.2	0.155	83
T_0_ dd-cfDNA (%)	0.2 ± 0.1	0.8 ± 1.7	2.0 ± 2.6	**<0.001**	106
T_0_ eGFR (mL/min/1.73m^2^)	33.4 ± 17.5	30.5 ± 16.4	24.8 ± 12.6	**0.004**	106
Immunology					
Preformed antibodies	19 (22.9)	9 (15.5)	10 (40.0)	**0.031**	83
T_0_ sCD30 (ng/mL)	33.9 ± 29.7	31.2 ± 26.7	39.0 ± 34.5	0.244	106
DSA >500 MFI	30 (29.4)	16 (23.5)	14 (41.2)	0.107	102
AB MM	2 ± 1	2 ± 1	2 ± 1	0.088	91
DR MM	1 ± 1	1 ± 1	1 ± 1	0.319	91
A/B/DR MM	2 ± 2	2 ± 2	3 ± 1	0.079	91
Immunosuppression					
Use of tacrolimus	82 (77.4)	52 (74.3)	30 (83.3)	0.418	106
Use of cyclosporine A	19 (17.9)	16 (22.9)	3 (8.3)	0.107	106
Use of mTOR inhibitors	7 (6.6)	6 (8.6)	1 (2.8)	0.418	106
Use of belatacept	3 (2.8)	1 (1.4)	2 (5.6)	0.266	106
Biopsy findings					
Acute rejection^a^	35 (33.0)	17 (24.3)	18 (50.0)	**0.0400**	106
Borderline changes	23 (21.7)	13 (18.6)	10 (27.8)	0.4600	106
AMR	7 (6.6)	2 (2.9)	5 (13.9)	**0.043**	106
TCMR	5 (4.7)	2 (2.9)	3 (8.3)	0.334	106
BKVAN	13 (12.3)	12 (17.1)	1 (2.8)	0.056	106
Follow-up data					
T_1_ dd-cfDNA (%)	0.5 ± 0.7	0.2 ± 0.2 (59)	1.1 ± 0.9 (32)	**<0.001**	91
T_2_ dd-cfDNA (%)	0.6 ± 0.8	0.3 ± 0.7 (50)	0.9 ± 0.9 (29)	**0.003**	79
T_3_ dd-cfDNA (%)	0.4 ± 0.8	0.3 ± 0.5 (51)	0.7 ± 1.1 (27)	0.079	78
Patient death	6 (5.7)	4 (5.7)	2 (5.6)	1.0	106
Indication for Re-Biopsy	21 (19.8)	12 (17.1)	9 (25.0)	0.482	106
Re-biopsy with rejection	7 (6.6)	4 (5.7)	3 (8.3)	0.687	106

For each variable, perecentages were calculated based on the number of participants with data available for that variable; observations with missing values were excluded from the denominator. Results are given as N (%) or mean ± SD. In case of incomplete follow-up, the number of analyzed patients is indicated in parentheses. Abbreviations: A/B/DR MM, human leukocyte antigen mismatch score; AMR, antibody-mediated rejection; ATI, acute tubular injury; BKVAN, BK virus-associated nephropathy; dd-cfDNA, donor-derived cell-free DNA; DSA, donor-specific antibodies; g/molCr, g/molCreatinine; MFI, mean fluorescence intensity; mTORi, mTOR inhibitor; PRA, panel reactive antibody; sCD30, soluble CD30; TCMR, T cell-mediated rejection; Tx, transplantation. T_0_ = at biopsy, T_1_ = 7 days post-biopsy, T_2_ = 30 days post-biopsy, T_3_ = 90 days post-biopsy

*P*-values less than 0.05 were considered statistically significant and are highlighted in **bold**; missing values were excluded; units/coding = measurement units or variable coding (continuous, categorical, %).

^a^ Rejection cases include patients with borderline changes.

**FIGURE 1:**
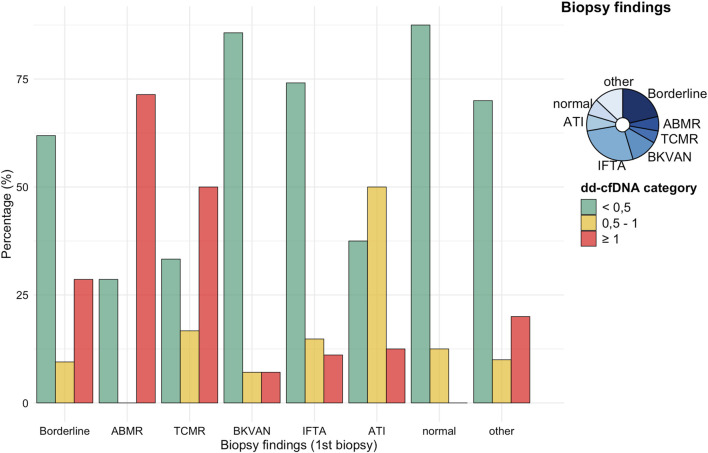
% dd-cfDNA in relation to histological diagnoses at initial biopsy. Bars indicate the proportion of patients with dd-cfDNA <0.5%, 0.5%–1.0%, and ≥1.0% for each biopsy category. The inset pie chart shows the overall distribution of biopsy findings. Borderline, n = 23; AMR, n = 7; TCMR, n = 6; BKVN, n = 13; IFTA, n = 29; ATI, n = 8; normal, n = 8; other, n = 14. Other changes included recurrent disease (*N* = 4), infection (*N* = 1), CNI toxicity (*N* = 1), or IFTA with signs of CNI toxicity (*N* = 8). AMR = antibody-mediated rejection; TCMR = T-cell–mediated rejection; BKVAN = BK virus nephropathy; IFTA = interstitial fibrosis/tubular atrophy; ATI = acute tubular injury; CNI = Calcineurin inhibitor toxicity.

Of the 108 allograft biopsies evaluated, 36/108 (33.3%) showed histological evidence of rejection. Among these, borderline changes were diagnosed in 23/108 (21.3%) cases, while AMR and T-cell-mediated rejection (TCMR) were identified in 7/108 (6.5%) and 6/108 (5.6%) biopsies, respectively (Figure 1). Most biopsies (72/108; 66.7%) showed no histological signs of rejection.

The distribution of dd-cfDNA levels (<0.5%, ≥0.5%, and ≥1.0%) differed notably across histopathological diagnoses at the time of indication biopsy (T_0_). At biopsy, dd-cfDNA was ≥0.5% in 38 cases (35.2%) and ≥1.0% or greater in 21 cases (19.4%). Among patients with AMR, the vast majority had dd-cfDNA levels ≥1.0%, while in patients with TCMR, most cases were associated with dd-cfDNA levels ≥0.5%, including a substantial fraction with levels ≥1.0%. Patients with borderline changes showed dd-cfDNA levels across all categories, but most were associated with values <0.5%. Histopathological diagnoses such as BK virus-associated nephropathy (BKVAN in 13/108; 12.0%), interstitial fibrosis and tubular atrophy (IFTA in 29/108; 26.9%), acute tubular injury (ATI in 8/108; 7.4%), and unspecific histology (in 22/108; 20.4%) occurred almost exclusively in patients with dd-cfDNA <0.5%, with only isolated cases in the ≥0.5% or ≥1.0% groups (Figure 1).

**FIGURE 2:**
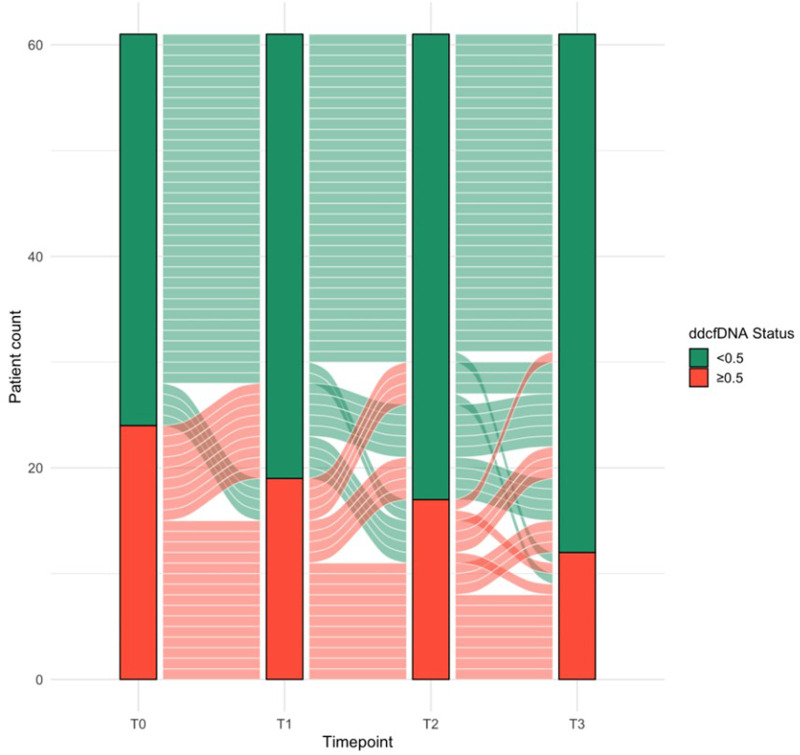
Alluvial diagram illustrating longitudinal trajectories of donor-derived cell-free DNA (dd-cfDNA) status from biopsy (T_0_) through follow-up (T_1_ = day 7, T_2_ = day 30, T_3_ = day 90). Bars show the proportion of patients above (≥0.5%, red) or below (<0.5%, green) the threshold at each timepoint; flow bands illustrate patient transitions between categories. Persistent versus resolving elevation is thereby visualized.

Figure 2 depicts the longitudinal dynamics of dd-cfDNA status (dichotomized at 0.5%) from the time of biopsy to 90 days post-biopsy. While most patients showed declining dd-cfDNA levels below 0.5% over time, a subset (n = 8) maintained persistently elevated levels across all measured timepoints. Among these, 4/8 (50%) showed progressive allograft dysfunction prompting a re-biopsy and 3/8 (37.5%) experienced graft failure (*P* = 0.0863 and *P* = 0.678, respectively). Conversely, patients whose dd-cfDNA normalized had more favorable clinical courses, with lower rates of re-biopsy (3/15; 20.0%) and graft failure (2/15; 13.3%).

### dd-cfDNA and Risk of eGFR Decline

Twenty-eight patients experienced a ≥30% decline in eGFR within 2 years following biopsy (28/106; 26.4%). Among these, 11/28 (39.3%) showed dd-cfDNA levels ≥0.5% and 8/28 (28.6%) had dd-cfDNA levels ≥1.0% at biopsy. Higher baseline dd-cfDNA predicted a faster decline in eGFR over the subsequent year (β = −2.91; *P* < 0.01).

Univariable Cox regression analyses (Table 2) showed that time-dependent dd-cfDNA was associated with ≥30% eGFR decline (HR 1.48; 95% CI 1.12–1.97; *P* = 0.006). Persistently elevated dd-cfDNA ≥0.5% (from biopsy to 90-day post-biopsy) showed a stronger association (HR 3.84; 95% CI 1.42–10.41; *P* = 0.0082), despite the small subgroup size (n = 8), whereas a >0.3% rise from biopsy to day 90 was not significant (*P* = 0.1170). Proteinuria at biopsy (HR 1.00 per g/mol creatinine; 95% CI 1.00–1.01; *P* = 0.0013) and acute rejection (HR 3.18; 95% CI 1.52–6.64; *P* = 0.0021), particularly AMR (HR 5.17; 95% CI 1.76–15.2; *P* = 0.0029), were also associated with eGFR decline.

**TABLE 2:** Univariate Cox Regression Analysis for ≥30% eGFR Decline within 2 years post-biopsy.

Variable	HR	95% CI	*P*-value
Demographics			
Recipient age (years)	1.00	0.97–1.02	0.7760
Male sex	1.18	0.56–2.5	0.6670
BMI >30 (kg/m^2^)	0.89	0.41–1.94	0.7710
Prior Tx	1.13	0.51–2.52	0.7650
Donor data			
Donor age	1.00	0.98–1.03	0.7860
Male donor	0.79	0.36–1.72	0.5490
Deceased donation	1.21	0.54–2.7	0.6400
Biopsy findings			
Acute rejection^a^	3.18	1.52–6.64	**0.0021**
AMR	5.17	1.76–15.2	**0.0029**
TCMR	2.60	0.90–7.51	0.0781
Borderline changes	0.76	0.23–2.53	0.6590
BKVAN	0.86	0.30–2.47	0.7760
Immunology			
Preformed antibodies	0.59	0.17–2.05	0.4070
PRA **≥**30%	0.55	0.16–1.92	0.3500
T_0_ sCD30	1.01	0.99–1.03	0.2140
A/B/DR MM	1.07	0.84–1.36	0.6170
DSA >500 MFI	1.13	0.49–2.59	0.7680
*de novo* DSA	1.30	0.18–9.58	0.7980
Immunosuppression			
CNI	0.60	0.18–1.99	0.3990
mTORi	1.56	0.37–6.61	0.5430
Belatacept	2.37	0.72–7.87	0.1580
Laboratory			
T_0_ eGFR (mL/min/1.73m^2^)	0.98	0.95–1.01	0.1610
T_0_ proteinuria (g/molCr)	1.00	1.00–1.01	**0.0013**
T_0_ dd-cfDNA (%, continuous)	1.14	1.01–1.30	**0.0405**
T_0_ dd-cfDNA **≥**0.5%	1.67	0.79–3.54	0.1810
T_0_ dd-cfDNA **≥**1%	2.28	1.03–5.05	**0.0426**
Time-dependent dd-cfDNA % (T_0_-T_3_)	1.48	1.12–1.97	**0.006**
Persistently high dd-cfDNA **≥**0.5% (T_0_-T_3_)	3.84	1.42–10.41	**0.0082**
dd-cfDNA T_0_-T_3_ >0.3%	2.27	0.81–6.30	0.1170
Baseline creatinine	1.15	0.73–1.81	0.5480
Histopathology/Banff composite scores			
TCMR/TI (t + i + v)	1.06	0.84–1.32	0.641
AMR/MVI (g + ptc + c4d)	1.30	0.96–1.77	0.094
Chronicity (ci + ct + cv + cg)	1.16	1.01–1.32	**0.030**

Abbreviations: A/B/DR MM, human leukocyte antigen mismatch score; AMR, antibody-mediated rejection; BKVAN, BK virus-associated nephropathy; BMI, body-mass index; CI, confidence interval; CNI, calcineurin inhibitor; dd-cfDNA, donor-derived cell-free DNA; DSA, donor-specific antibodies; g/molCr, g/mol Creatinine; HR, hazard ratio; MFI, mean fluorescence intensity; mTORi, mTOR, inhibitor; PRA, panel reactive antibody; sCD30, soluble CD30; TCMR, T cell-mediated rejection; Tx, transplantation. T_0_ = at biopsy, T_1_ = 7 days post-biopsy, T_2_ = 30 days post-biopsy, T_3_ = 90 days post-biopsy. Histopathology and Banff composite scores: Histological findings were assessed according to the Banff classification. To reduce collinearity and improve model stability, composite Banff domains were used in regression analyses. Banff composite domains were defined as: T-cell-mediated rejection (TCMR)/tubulointerstitial inflammation (TI) as t + i + v, where t = tubulitis, i = interstitial inflammation, v = intimal arteritis, antibody-mediated rejection (AMR)/microvascular inflammation (MVI) as g + ptc + c4d, where g = glomerulitis, ptc = peritubular capillaritis, c4d = C4d positivity, and chronicity as ci + ct + cv + cg, where ci = interstitial fibrosis, ct = tubular atrophy, cv = vascular fibrous intimal thickening, cg = transplant glomerulopathy. *P*-values less than 0.05 were considered statistically significant and are highlighted in **bold**; missing values were excluded; units/coding = measurement units or variable coding (continuous, categorical, %).

^a^ Rejection cases include patients with Borderline changes.

**FIGURE 3:**
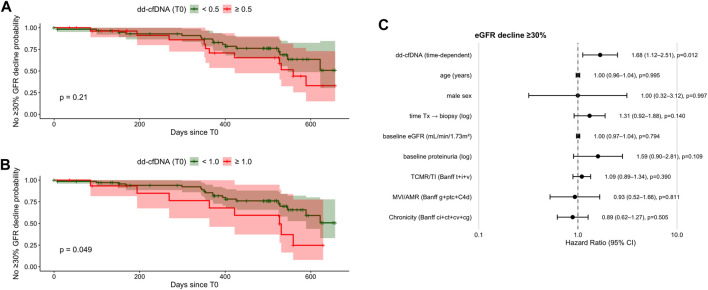
Kaplan–Meier curves and multivariate time-dependent Cox regression for ≥30% eGFR decline within 2 years post-biopsy. **(A)** Kaplan–Meier estimates comparing patients with dd-cfDNA <0.5% vs. ≥ 0.5% at biopsy (log-rank *P* = 0.18). **(B)** Kaplan–Meier estimates for dd-cfDNA <1.0% vs. ≥ 1.0% at biopsy (log-rank *P* = 0.04). **(C)** Forest plot from time-dependent multivariate Cox regression including dd-cfDNA as a continuous longitudinal variable and clinical covariates. Hazard ratios (HR) with 95% confidence intervals (CI) are shown. dd-cfDNA, donor-derived cell-free DNA; eGFR, estimated glomerular filtration rate; HR, hazard ratio; CI, confidence interval; Tx, transplantation.

When analyzing single measurements at biopsy, the 0.5% cutoff showed no significant association with eGFR decline (Table 2; Figure 3A). In contrast, single measurements at biopsy, both as a continuous variable (HR 1.14; 95% CI 1.01–1.30; *P* = 0.0405) and using the ≥1.0% cutoff (HR 2.28; 95% CI 1.03–5.05; *P* = 0.0426), were predictive of risk for eGFR decline (Table 2; Figure 3B).

In the multivariable time-dependent Cox model adjusting for age, sex, time from transplantation to biopsy, baseline eGFR, baseline proteinuria, and histological injury summarized by Banff domains, longitudinal dd-cfDNA remained independently associated with a ≥30% decline in eGFR within 2 years (HR 1.68; 95% CI 1.12–2.51; *P* = 0.012; Figure 3C). None of the prespecified Banff domain scores were independently associated with eGFR decline after adjustment.

### dd-cfDNA and Risk for Progressive Allograft Dysfunction Prompting Re-Biopsy

Twenty-one patients (21/106; 19.8%) underwent re-biopsy at a median of 299 days (IQR 112-752) post-initial biopsy. In univariate analysis (Table 3), dd-cfDNA at biopsy was associated with re-biopsy (HR 1.24; 95% CI 1.06–1.46; *P* = 0.0091), and persistently elevated dd-cfDNA ≥0.5% (from biopsy to 90-day post-biopsy) showed a borderline association (HR 3.15; 95% CI 0.99–10.01; *P* = 0.0521). Other laboratory values at the time of biopsy, including eGFR and proteinuria, were not associated with a re-biopsy. TCMR at index biopsy (HR 6.13; 95% CI 1.70–24.7; *P* < 0.001), higher baseline creatinine (HR 1.80; 95% CI 1.04–1.96; *P* = 0.0066), and preformed antibodies (HR 2.63; 95% CI 1.01–6.84; *P* = 0.0483), however, were also associated with the indication for re-biopsy.

**TABLE 3:** Univariate cox regression analysis for progressive allograft dysfunction requiring repeat biopsy.

Variable	HR	95% CI	*P*-value
Demographics			
Recipient age (years)	0.98	0.95–1.01	0.1520
Male sex	0.76	0.29–1.96	0.5670
BMI >30 (kg/m^2^)	0.20	0.03–1.46	0.1110
Prior Tx	1.42	0.69–2.90	0.3420
Donor data			
Donor age	1.03	1.00–1.06	0.0890
Male donor	0.51	0.21–1.28	0.1520
Deceased donation	1.17	0.47–2.92	0.7380
Biopsy findings			
Acute rejection^a^	2.13	0.90–5.02	0.0850
AMR	0.00	0.00-lnf	0.9970
TCMR	6.13	1.70–24.70	**<0.001**
Borderline changes	1.27	0.42–3.82	0.6670
BKVAN	1.71	0.58–5.10	0.3340
Immunology			
Preformed antibodies	2.63	1.01–6.84	**0.0483**
PRA **≥** 30%	2.33	0.89–6.07	0.0839
T_0_ sCD30	1.00	0.99–1.02	0.636
A/B/DR MM	0.89	0.68–1.16	0.3760
DSA >500 MFI	0.44	0.13–1.51	0.1940
*de novo* DSA	2.41	0.32–18.41	0.393
Immunosuppression			
CNI	1.44	0.19–10.73	0.7230
mTORi	1.14	0.15–8.58	0.8360
Belatacept	0.92	0.12–6.82	0.9310
Laboratory			
T_0_ eGFR (mL/min/1.73m^2^)	0.97	0.94–1.00	0.0828
T_0_ proteinuria (g/molCr)	1.00	1.00–1.00	0.6200
T_0_ dd-cfDNA (%, continuous)	1.24	1.06–1.46	**0.0091**
T_0_ dd-cfDNA **≥**0.5%	1.58	0.66–3.76	0.3000
T_0_ dd-cfDNA **≥**1%	1.21	0.44–3.30	0.7130
Time-dependent dd-cfDNA % (T_0_-T_3_)	1.63	1.33–2.00	**<0.001**
Persistently high dd-cfDNA **≥**0.5% (T_0_-T_3_)	3.15	0.99–10.01	0.0521
dd-cfDNA T_0_-T_3_ >0.3%	0.95	0.35–2.58	0.9230
Baseline creatinine	1.80	1.04–1.96	**0.0066**
Histopathology/Banff composite scores			
TCMR/TI (t + i + v)	1.23	0.96–1.57	0.103
AMR/MVI (g + ptc + c4d)	1.04	0.67–1.63	0.853
Chronicity (ci + ct + cv + cg)	0.92	0.77–1.10	0.354

Abbreviations: A/B/DR MM, human leukocyte antigen mismatch score; AMR, antibody-mediated rejection; BKVAN, BK virus-associated nephropathy; BMI, body-mass index; CI, confidence interval; CNI, calcineurin inhibitor; dd-cfDNA, donor-derived cell-free DNA; DSA, donor-specific antibodies; g/molCr, g/mol Creaatinine; HR, hazard ratio; MFI, mean fluorescence intensity; mTORi, mTOR, inhibitor; PRA, panel reactive antibody; sCD30, soluble CD30; TCMR, T cell-mediated rejection; Tx, transplantation. T_0_ = at biopsy, T_1_ = 7 days post-biopsy, T_2_ = 30 days post-biopsy, T_3_ = 90 days post-biopsy. Histopathology and Banff composite scores: Histological findings were assessed according to the Banff classification. To reduce collinearity and improve model stability, composite Banff domains were used in regression analyses. Banff composite domains were defined as: T-cell-mediated rejection (TCMR)/tubulointerstitial inflammation (TI) as t + i + v, where t = tubulitis, i = interstitial inflammation, v = intimal arteritis, antibody-mediated rejection (AMR)/microvascular inflammation (MVI) as g + ptc + c4d, where g = glomerulitis, ptc = peritubular capillaritis, c4d = C4d positivity, and chronicity as ci + ct + cv + cg, where ci = interstitial fibrosis, ct = tubular atrophy, cv = vascular fibrous intimal thickening, cg = transplant glomerulopathy.

P-values less than 0.05 were considered statistically significant and are highlighted in **bold**; missing values were excluded; units/coding = measurement units or variable coding (continuous, categorical, %).

^a^ Rejection cases include patients with Borderline changes.

**FIGURE 4:**
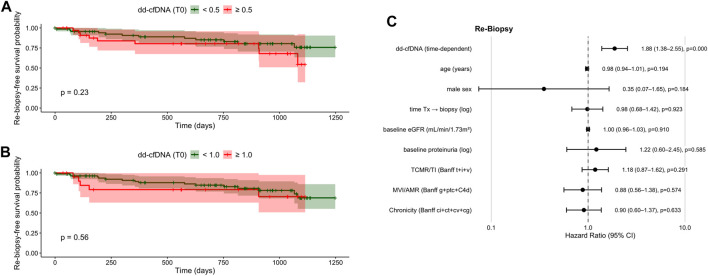
Kaplan–Meier curves and multivariate, time-dependent Cox regression for re-biopsy–free survival after kidney transplantation. **(A)** Kaplan–Meier estimates stratified by dd-cfDNA <0.5% vs. ≥ 0.5% at biopsy (log-rank *P* = 0.23). **(B)** Kaplan–Meier estimates stratified by dd-cfDNA <1.0% vs. ≥ 1.0% at biopsy (log-rank *P* = 0.56). **(C)** Forest plot of time-dependent multivariate Cox regression including dd-cfDNA as a continuous longitudinal variable and clinical covariates. Hazard ratios (HR) with 95% confidence intervals (CI) are shown. dd-cfDNA, donor-derived cell-free DNA; eGFR, estimated glomerular filtration rate; HR, hazard ratio; CI, confidence interval; Tx, transplantation.

By contrast, single-timepoint cutoffs at biopsy using both the ≥0.5% and ≥1.0% cutoffs were not significantly associated with progressive allograft dysfunction prompting a re-biopsy (Figures 4A, B).

When longitudinal dd-cfDNA was entered into the prespecified multivariable time-dependent Cox model together with age, sex, time from transplantation to biopsy, baseline eGFR, baseline proteinuria, and aggregated Banff domain scores, higher dd-cfDNA levels were independently associated with an increased likelihood of re-biopsy due to progressive allograft dysfunction (HR 1.88; 95% CI 1.38–2.55; P < 0.001; Figure 4C). In contrast, histological injury domains did not retain independent prognostic significance after adjustment.

### dd-cfDNA and Risk of Graft Failure

Graft failure occurred in 24/106 (22.6%) kidney transplant recipients with indication biopsy at a median of 624 days post-biopsy (IQR 336–829) and at a median of 1739 days post-transplant (IQR 1074–4,281).

**FIGURE 5:**
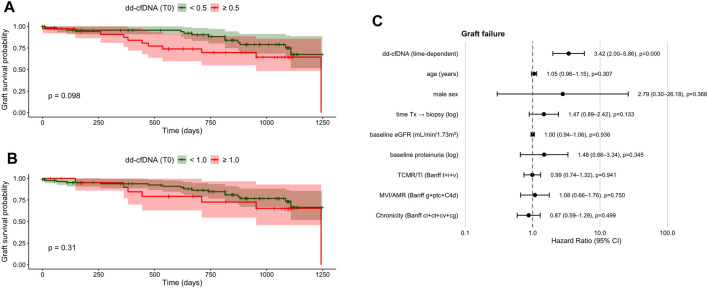
Kaplan–Meier curves and multivariate time-dependent Cox regression for graft survival after kidney transplantation. **(A)** Kaplan–Meier estimates stratified by dd-cfDNA <0.5% vs. ≥ 0.5% at biopsy (log-rank *P* = 0.098). **(B)** Kaplan–Meier estimates stratified by dd-cfDNA <1.0% vs. ≥ 1.0% at biopsy (log-rank *P* = 0.31). **(C)** Forest plot from time-dependent multivariate Cox regression including dd-cfDNA as a continuous longitudinal variable and clinical covariates. Hazard ratios (HR) with 95% confidence intervals (CI) are shown. dd-cfDNA, donor-derived cell-free DNA; eGFR, estimated glomerular filtration rate; HR, hazard ratio; CI, confidence interval; Tx, transplantation.

Single-timepoint dd-cfDNA cutoffs at biopsy (≥0.5% and ≥1.0%) were not significantly associated with risk of graft failure (Table 4; Figures 5A, B). However, a non-significant trend toward increased risk of graft failure was observed in patients with dd-cfDNA levels ≥0.5% compared to those with levels <0.5% (28.9% vs. 18.6%, *P* = 0.098 Figure 5A). In univariate Cox regression analysis (Table 4), demographic variables were not associated with graft failure. Time-dependent dd-cfDNA (biopsy to day 90) was associated with higher graft failure risk (HR 1.51; 95% CI 1.11–2.04; *P* = 0.0080), while persistently elevated dd-cfDNA levels ≥0.5% showed a non-significant trend (HR 2.94; 95% CI 0.92–9.44; *P* = 0.0700). Baseline serum creatinine was also significantly associated with graft failure (HR 1.68; 95% CI 1.04–2.71; *P* = 0.0339). Among biopsy findings, only TCMR was significant (HR 3.93; 95% CI 1.15–13.38; *P* = 0.0287), whereas acute rejection overall and AMR were not. Other immunological markers such as preformed antibodies, panel reactive antibodies ≥30%, and sCD30 levels, as well as donor characteristics or immunosuppressive treatments showed no significant association with graft failure.

**TABLE 4:** Univariate Cox Regression analysis for Graft Failure.

Variable	HR	95% CI	*P*-value
Demographics			
Recipient age (years)	1.01	0.98–1.04	0.5170
Male sex	1.68	0.75–3.76	0.2070
Prior Tx	1.09	0.53–2.27	0.8100
BMI >30 (kg/m^2^)	0.19	0.03–1.39	0.1020
Donor data			
Donor age	1.02	0.99–1.05	0.2440
Male donor	0.68	0.29–1.57	0.3680
Deceased donation	1.34	0.55–3.29	0.5230
Biopsy findings			
Acute rejection^a^	1.32	0.58–2.97	0.5060
AMR	2.97	0.68–12.98	0.1480
TCMR	3.93	1.15–13.38	**0.0287**
Borderline changes	0.38	0.09–1.64	0.1940
BKVAN	1.11	0.33–3.75	0.8640
Immunology			
Preformed antibodies	1.17	0.37–3.67	0.7920
PRA **≥**30%	1.29	0.40–4.20	0.6670
T_0_ sCD30	1.01	0.99–1.02	0.2080
A/B/DR MM	1.05	0.8–1.38	0.7400
DSA >500 MFI	0.82	0.32–2.07	0.6670
*de novo* DSA	1.71	0.23–12.77	0.6000
Immunosuppression			
CNI	0.70	0.16–2.99	0.6260
mTORi	1.90	0.44–8.14	0.4200
Belatacept	1.94	0.45–8.34	0.3740
Laboratory			
T_0_ eGFR (mL/min/1.73m^2^)	0.98	0.95–1.00	0.0965
T_0_ proteinuria (g/molCr)	1.00	1.00–1.00	0.0852
T_0_ dd-cfDNA (%, continuous)	1.66	1.20–2.29	**0.002**
T_0_ dd-cfDNA **≥**0.5%	1.83	0.83–4.40	0.1280
T_0_ dd-cfDNA **≥**1%	1.43	0.59–3.49	0.4270
Time-dependent dd-cfDNA % (T_0_-T_3_)	1.51	1.11–2.04	**0.0080**
Persistently high dd-cfDNA **≥**0.5% (T_0_-T_3_)	2.94	0.92–9.44	0.0700
dd-cfDNA T_0_-T_3_ >0.3%	3.56	0.8–15.79	0.0965
Baseline creatinine	1.68	1.04–2.71	**0.0339**
Histopathology/Banff composite scores			
TCMR/TI (t + i + v)	1.01	0.77–1.34	**0.921**
AMR/MVI (g + ptc + c4d)	1.23	0.84–1.81	**0.295**
Chronicity (ci + ct + cv + cg)	1.21	1.04–1.42	**0.295**

Abbreviations: A/B/DR MM, human leukocyte antigen mismatch score; AMR, antibody-mediated rejection; BKVAN, BK, virus-associated nephropathy; BMI, body-mass index; CI, confidence interval; CNI, calcineurin inhibitor; dd-cfDNA, donor-derived cell-free DNA; DSA, donor-specific antibodies; g/molCr, g/molCreatinine; HR, hazard ratio; MFI, mean fluorescence intensity; mTORi, mTOR, inhibitor; PRA, panel reactive antibody; sCD30, soluble CD30; TCMR, T cell-mediated rejection; Tx, transplantation. T_0_ = at biopsy, T_1_ = 7 days post-biopsy, T_2_ = 30 days post-biopsy, T_3_ = 90 days post-biopsy. Histopathology and Banff composite scores: Histological findings were assessed according to the Banff classification. To reduce collinearity and improve model stability, composite Banff domains were used in regression analyses. Banff composite domains were defined as: T-cell-mediated rejection (TCMR)/tubulointerstitial inflammation (TI) as t + i + v, where t = tubulitis, i = interstitial inflammation, v = intimal arteritis, antibody-mediated rejection (AMR)/microvascular inflammation (MVI) as g + ptc + c4d, where g = glomerulitis, ptc = peritubular capillaritis, c4d = C4d positivity, and chronicity as ci + ct + cv + cg, where ci = interstitial fibrosis, ct = tubular atrophy, cv = vascular fibrous intimal thickening, cg = transplant glomerulopathy.

P-values less than 0.05 were considered statistically significant and are highlighted in **bold**; missing values were excluded; units/coding = measurement units or variable coding (continuous, categorical, %).

^a^ Rejection cases include patients with Borderline changes.

In adjusted time-dependent Cox analysis accounting for demographic factors, time from transplantation to biopsy, baseline graft function, baseline proteinuria, and Banff domain-based histological injury burden, longitudinal dd-cfDNA emerged as a strong independent predictor of graft failure (HR 3.42; 95% CI 2.00–5.86; P < 0.001; Figure 5C). None of the histological domain scores were independently associated with graft loss in the fully adjusted model.

## Discussion

In this longitudinal study of 106 kidney transplant recipients undergoing indication biopsies with concurrent dd-cfDNA measurement, we demonstrated that elevated donor-derived dd-cfDNA levels were strongly associated with adverse allograft outcomes, including subsequent eGFR decline, persistent allograft dysfunction requiring re-biopsy, and eventual allograft failure. These associations were most pronounced when dd-cfDNA was modeled as a time-dependent variable, with longitudinal trajectories capturing additional prognostically relevant information beyond single measurements.

Evidently, measured at a single time point, dd-cfDNA is very informative for identifying ongoing allograft injury, with high levels being primarily indicative for rejection [7, 18, 25, 26]. Consistent with these findings, dd-cfDNA levels in our cohort at time of biopsy were highest in patients with AMR [median (IQR) 2.00% (0.48–3.20)] and TCMR [median (IQR) 0.92% (0.19–11.25)] [4]. Notably, however, elevations in dd-cfDNA may also occur in other settings, such as ischemia-reperfusion injury (IRI), acute parenchymal injury seen with acute kidney injury, and sometimes severe cases of BKVAN [27, 28]. Interestingly though, even in these non-rejection settings, persistently elevated dd-cfDNA appears prognostically relevant: Cucchiari et al. showed for instance that early post-transplant dd-cfDNA kinetics reflected IRI severity as higher levels at 24 h and persistent elevation at day 7 were associated with delayed graft function, lower 6-month eGFR, and worse iBox-estimated 7-year graft survival, whereas normalization to <0.5% within the first week predicted better outcomes [29].

We also demonstrated that patients with dd-cfDNA ≥1.0% at biopsy more often presented with lower baseline eGFR, reflecting the clinical reality that conventional markers often prompt biopsy only once graft injury is already advanced. Declining eGFR or increasing proteinuria are late indicators of damage and frequently leave only limited therapeutic options once detected. In contrast, dd-cfDNA may provide earlier insight, as shown by Bromberg et al., who reported that dd-cfDNA elevations can precede biopsy-triggering changes in creatinine or proteinuria by several months, including up to 5 months before AMR diagnosis [5]. Interestingly, they also reported that elevated dd-cfDNA in non-rejecting patients was associated with decreased eGFR, suggesting that dd-cfDNA may capture broader forms of allograft injury beyond histological rejection and may serve as an early marker of functional decline. Interestingly, patients with “high immunological risk”, such as those with *de novo* DSA, re-transplantation status, or C4d-positive biopsies, frequently had two or more consecutive dd-cfDNA measurements ≥1% [5], matching our data that patients with dd-cfDNA ≥1.0% at biopsy more often had preformed antibodies, and histopathological evidence of AMR, consistent with ongoing immune-mediated injury. It appears as if precisely these patients may be just subthreshold rejection. Even without meeting full diagnostic criteria, evidence suggests that they are already posed at risk and warrant close monitoring, a situation in which dd-cfDNA may help monitor graft injury and identify a critical window for therapeutic intervention before irreversible chronic damage occurs [5].

Building on the association between elevated dd-cfDNA at biopsy and adverse outcomes observed in our cohort and reported in prior studies [18, 21, 22], our longitudinal analysis further demonstrates that persistent dd-cfDNA elevation over time may also carry important prognostic significance, supporting the utility of serial monitoring. Specifically, longitudinal dd-cfDNA trajectories were independently associated with ≥30% eGFR decline, the need for re-biopsy, and graft failure, even after accounting for histological injury at the time of biopsy. This suggests dd-cfDNA reflects ongoing injury that may not be fully captured by a single biopsy, especially in patients with evolving damage. Among patients with persistently high levels, half required re-biopsies and more than one-third lost their graft. These findings align with data from the ADMIRAL trial, where elevated dd-cfDNA (≥0.5%) was associated with a near threefold higher risk of *de novo* DSA development and persistent elevation with an almost twofold higher risk of >25% eGFR decline over 3 years [18]. Our results also parallel those of Bromberg et al., who linked sustained high dd-cfDNA-elevation to poor outcomes irrespective of histology [5], and of Bunnapradist et al., who demonstrated that dd-cfDNA trends following rejection were strongly associated with subsequent rejection or allograft dysfunction [21].

Notably, most biopsied patients (66.7%) in our cohort showed no histopathological signs of rejection, with correspondingly low dd-cfDNA levels in this subgroup. This naturally raises the question whether all such patients require a biopsy. Insights into this issue may be drawn from the multicenter Kidney Allograft Outcomes AlloSure Registry (KOAR) study, which reported a significantly higher rejection yield when dd-cfDNA was elevated (defined as a level ≥1% or dd-cfDNA ≥0.5% with a ≥61% increase from the prior test), with 39% vs. 7% rejection cases in the surveillance setting and 47% vs. 12% in the for-cause setting (*P* < 0.001) [30]. These results indicate that elevated dd-cfDNA may meaningfully improve the pre-test probability of histological rejection and the diagnostic yield of a biopsy, helping to identify patients most likely to benefit from histological assessment. This should not be interpreted as an argument against biopsy *per se*, as histology may still reveal other actionable findings such as calcineurin inhibitor toxicity, where therapeutic adjustments may still improve graft outcomes.

Taken together, our data indicate that repeated dd-cfDNA monitoring may provide complementary prognostic information beyond traditional markers such as allograft function, proteinuria, and histopathology. Consistent results from both single-center and multicenter studies position dd-cfDNA as a robust biomarker of graft injury [22, 31–33]. In clinical context, dd-cfDNA may help inform follow-up strategies and decisions about when to consider re-biopsy. However, realizing this potential clinical utility critically depends on appropriate patient selection. Patients with DSA or a history of rejection may particularly benefit from targeted dd-cfDNA surveillance, including monitoring AMR activity following anti-rejection therapy [2, 34, 35]. By contrast, in low-risk recipients with stable graft function, isolated dd-cfDNA elevations are often transient and not consistently associated with adverse outcomes, suggesting limited value for routine surveillance in this group [36]. These observations also align with the broader debate summarized by Naesens and Wong, emphasizing that the clinical utility of dd-cfDNA depends mainly on predictive values and likelihood ratios, which are strongly influenced by pre-test probability. The value of dd-cfDNA testing is therefore greatest in settings with a high prevalence of rejection, such as in patients with de novo DSA, where a positive result can raise the probability of rejection from ∼50% to ∼75%. By contrast, in low-prevalence, stable populations its impact is limited and false-positive results may potentially trigger unnecessary biopsies [37].

The strengths of our study include its longitudinal design with repeated measurements, integration of histological, immunological, and functional data, and use of time-dependent Cox regression to account for intra-individual variability. Our real-world, heterogeneous cohort reflects clinical practice. The main limitations of this study are the modest number of events and limited numbers in certain subgroups, leading to uncertainty and wide confidence intervals for some estimates. In addition, dd-cfDNA was not available at every follow-up time point for all patients, which may limit some longitudinal analyses. Further, indication for re-biopsy is a clinician-driven endpoint, likely influenced by surveillance intensity and local practice, and should therefore be interpreted cautiously. Notably, strongest associations were observed for ≥30% eGFR decline and graft failure, which are less susceptible to indication bias and more directly reflect long term allograft prognosis. Finally, all analyses are exploratory and hypothesis-generating, intended to describe longitudinal patterns rather than providing definitive risk estimates. Larger multicenter cohorts with higher event rates are needed to obtain more precise estimates, to formally assess interactions, and to validate our observed associations.

Before our findings can be translated into practice, several important questions remain, including how dd-cfDNA can best be integrated into routine care, how often it should be measured, and whether decisions on clinical intervention should rely not only on absolute thresholds but also on patient-specific changes over time, as recently also outlined by the STAR working group [38]. The dd-cfDNA thresholds used in this study were chosen to align with commonly used clinical cutoffs and ensure comparability with existing literature, but although validated for detecting acute rejection, they were not specifically developed for prognostic assessment of long-term graft function. Future studies should therefore establish validated, outcome-specific (and potentially time-dependent) thresholds to support interpretation of dd-cfDNA in routine care. Further, regulatory hurdles persist, particularly the lack of cost-effectiveness analyses that clarify to what extent dd-cfDNA monitoring can reduce biopsies or improve their timing to secure the best therapeutic window [37, 39]. Comparative data between different assays, which could help drive competition and incentivize more cost-effective testing, are also scarce. Addressing these challenges will be essential to fully realize the promise of dd-cfDNA as a clinically meaningful tool that not only informs diagnosis and prognosis but also improves long-term care in kidney transplantation.

## Data Availability

The raw data supporting the conclusions of this article will be made available by the authors, without undue reservation.
