# Supplementary material for: Longitudinal Monitoring of Donor-Derived Cell-Free DNA Supports Risk Stratification in Kidney Transplant Recipients With Allograft Dysfunction
Source: Transpl Int. 2026 Mar 12;39:15929. doi: 10.3389/ti.2026.15929 (PMC13017682; doi:10.3389/ti.2026.15929)
Supplement: Supplementary file 1 [file Supplementaryfile1.docx]

# Supplementary Material – Table of Contents

# Supplementary Methods

# Supplementary Tables S1–S6: Outcome A – ≥30% decline in eGFR:

# primary model, proportional hazards test, sensitivity analysis (no histology) with proportional hazards test, and sensitivity analysis (chronicity domain only) with proportional hazards test.

# Supplementary Tables S7–S12: Outcome B – Re-biopsy:

# primary model, proportional hazards test, sensitivity analysis (no histology) with proportional hazards test, and sensitivity analysis (chronicity domain only) with proportional hazards test.

# Supplementary Tables S13–S18: Outcome C – Graft failure:

primary model, proportional hazards test, sensitivity analysis (no histology) with proportional hazards test, and sensitivity analysis (chronicity domain only) with proportional hazards test.Supplementary Methods

# Supplementary Methods

Time-dependent Cox regression models were fitted for three outcomes using start–stop intervals for repeated dd-cfDNA measurements. Robust standard errors were calculated with clustering by patient ID. Banff composite domains were defined as: T-cell-mediated rejection (TCMR)/tubulointerstitial inflammation (TI) as t+i+v, where t = tubulitis, i = interstitial inflammation, v = intimal arteritis, antibody-mediated rejection (AMR)/microvascular inflammation (MVI) as g+ptc+c4d, where g = glomerulitis, ptc = peritubular capillaritis, c4d = C4d positivity, and chronicity as ci+ct+cv+cg, where ci = interstitial fibrosis, ct = tubular atrophy, cv = vascular fibrous intimal thickening, cg = transplant glomerulopathy

# Outcome A: ≥30% decline in eGFR

**Model summary:** C-index=0.717 (SE 0.062); LR p=0.20; Wald p=0.002; Score p=0.07; Robust score p=0.20; Global PH p=0.33.

## Supplementary Table S1. Outcome A – Primary model

| **Variable** | **HR** | **Robust SE** | **p-value** | **95% CI** |
| --- | --- | --- | --- | --- |
| Time-dependent dd-cfDNA | 1.68 | 0.206 | 0.012 | 1.1195–2.5120 |
| Recipient age (years) | 1.00 | 0.021 | 0.995 | 0.9592–1.0430 |
| Male recipient sex | 1.00 | 0.582 | 0.997 | 0.3193–3.1200 |
| Time from transplantation to biopsy (log-transformed) | 1.31 | 0.184 | 0.14 | 0.9150–1.8800 |
| Baseline eGFR (mL/min/1.73 m²) | 1.01 | 0.019 | 0.794 | 0.9683–1.0430 |
| Baseline proteinuria (log-transformed) | 1.59 | 0.290 | 0.109 | 0.9019–2.8150 |
| TCMR/TI (t+i+v) | 1.09 | 0.105 | 0.39 | 0.8908–1.3450 |
| AMR/MVI (g+ptc+c4d) | 0.93 | 0.294 | 0.811 | 0.5233–1.6600 |
| Chronicity (ci+ct+cv+cg) | 0.89 | 0.183 | 0.505 | 0.6187–1.2670 |

## Supplementary Table S2. Outcome A – Proportional hazards test (primary model)

| **Variable** | **PH test p-value** |
| --- | --- |
| Time-dependent dd-cfDNA | 0.421 |
| Recipient age (years) | 0.37 |
| Sex | 0.1 |
| Time from transplantation to biopsy (log-transformed) | 0.424 |
| Baseline eGFR (mL/min/1.73 m²) | 0.091 |
| Baseline proteinuria (log-transformed) | 0.252 |
| TCMR/TI (t+i+v) | 0.342 |
| AMR/MVI (g+ptc+c4d) | 0.28 |
| Chronicity (ci+ct+cv+cg) | 0.92 |
| GLOBAL | 0.33 |

**Model summary:** C-index=0.739 (SE 0.063); LR p=0.08; Wald p=0.003; Score p=0.02; Robust score p=0.09; Global PH p=0.31.

## Supplementary Table S3. Outcome A – Sensitivity analysis (no histology)

| **Variable** | **HR** | **Robust SE** | **p-value** | **95% CI** |
| --- | --- | --- | --- | --- |
| Time-dependent dd-cfDNA | 1.65 | 0.212 | 0.018 | 1.0898–2.5030 |
| Recipient age (years) | 1.00 | 0.020 | 0.952 | 0.9601–1.0390 |
| Male recipient sex | 0.90 | 0.601 | 0.866 | 0.2783–2.9340 |
| Time from transplantation to biopsy (log-transformed) | 1.19 | 0.135 | 0.198 | 0.9134–1.5490 |
| Baseline eGFR (mL/min/1.73 m²) | 1.01 | 0.020 | 0.64 | 0.9710–1.0490 |
| Baseline proteinuria (log-transformed) | 1.41 | 0.223 | 0.121 | 0.9129–2.1870 |

## Supplementary Table S4. Outcome A – Proportional hazards test (no histology)

| **Variable** | **PH test p-value** |
| --- | --- |
| Time-dependent dd-cfDNA | 0.647 |
| Recipient age (years) | 0.522 |
| Sex | 0.146 |
| Time from transplantation to biopsy (log-transformed) | 0.465 |
| Baseline eGFR (mL/min/1.73 m²) | 0.078 |
| Baseline proteinuria (log-transformed) | 0.267 |
| GLOBAL | 0.31 |

**Model summary:** C-index=0.717 (SE 0.065); LR p=0.09; Wald p=0.002; Score p=0.03; Robust score p=0.10; Global PH p=0.43.

## Supplementary Table S5. Outcome A – Sensitivity analysis (chronicity domain only)

| **Variable** | **HR** | **Robust SE** | **p-value** | **95% CI** |
| --- | --- | --- | --- | --- |
| Time-dependent dd-cfDNA | 1.75 | 0.167 | <0.001 | 1.2591–2.4250 |
| Recipient age (years) | 1.00 | 0.021 | 0.966 | 0.9600–1.0430 |
| Male recipient sex | 0.96 | 0.593 | 0.938 | 0.2988–3.0540 |
| Time from transplantation to biopsy (log-transformed) | 1.32 | 0.151 | 0.067 | 0.9805–1.7710 |
| Baseline eGFR (mL/min/1.73 m²) | 1.01 | 0.019 | 0.696 | 0.9705–1.0460 |
| Baseline proteinuria (log-transformed) | 1.59 | 0.288 | 0.108 | 0.9033–2.7980 |
| Chronicity (ci+ct+cv+cg) | 0.85 | 0.145 | 0.25 | 0.6369–1.1250 |

## Supplementary Table S6. Outcome A – Proportional hazards test (chronicity only)

| **Variable** | **PH test p-value** |
| --- | --- |
| Time-dependent dd-cfDNA | 0.43 |
| Recipient age (years) | 0.422 |
| Sex | 0.143 |
| Time from transplantation to biopsy (log-transformed) | 0.438 |
| Baseline eGFR (mL/min/1.73 m²) | 0.096 |
| Baseline proteinuria (log-transformed) | 0.24 |
| Chronicity (ci+ct+cv+cg) | 0.886 |
| GLOBAL | 0.43 |

# Outcome B: Re-biopsy

**Model summary:** Interval rows n=268; Events=12; C-index=0.762 (SE 0.067); LR p=0.30; Wald p=2e-05; Score p=0.08; Robust score p=0.60; Global PH p=0.15.

## Supplementary Table S7. Outcome B – Primary model

| **Variable** | **HR** | **Robust SE** | **p-value** | **95% CI** |
| --- | --- | --- | --- | --- |
| Time-dependent dd-cfDNA | 1.88 | 0.156 | 5.39e-05 | 1.3831–2.5500 |
| Recipient age (years) | 0.98 | 0.018 | 0.194 | 0.9421–1.0120 |
| Male recipient sex | 0.35 | 0.790 | 0.184 | 0.0744–1.6460 |
| Time from transplantation to biopsy (log-transformed) | 0.98 | 0.188 | 0.923 | 0.6795–1.4190 |
| Baseline eGFR (mL/min/1.73 m²) | 1.00 | 0.018 | 0.91 | 0.9639–1.0330 |
| Baseline proteinuria (log-transformed) | 1.22 | 0.358 | 0.585 | 0.6025–2.4540 |
| TCMR/TI (t+i+v) | 1.18 | 0.159 | 0.291 | 0.8660–1.6170 |
| AMR/MVI (g+ptc+c4d) | 0.88 | 0.228 | 0.574 | 0.5627–1.3760 |
| Chronicity (ci+ct+cv+cg) | 0.90 | 0.211 | 0.633 | 0.5985–1.3670 |

## Supplementary Table S8. Outcome B – Proportional hazards test (primary model)

| **Variable** | **PH test p-value** |
| --- | --- |
| Time-dependent dd-cfDNA | 0.82 |
| Recipient age (years) | 0.32 |
| Sex | 0.21 |
| Time from transplantation to biopsy (log-transformed) | 0.45 |
| Baseline eGFR (mL/min/1.73 m²) | 0.24 |
| Baseline proteinuria (log-transformed) | 0.2 |
| TCMR/TI (t+i+v) | 0.25 |
| AMR/MVI (g+ptc+c4d) | 0.67 |
| Chronicity (ci+ct+cv+cg) | 0.51 |
| GLOBAL | 0.15 |

**Model summary:** C-index=0.775 (SE 0.053); LR p=0.20; Wald p=0.006; Score p=0.04; Robust score p=0.40; Global PH p=0.29.

## Supplementary Table S9. Outcome B – Sensitivity analysis (no histology)

| **Variable** | **HR** | **Robust SE** | **p-value** | **95% CI** |
| --- | --- | --- | --- | --- |
| Time-dependent dd-cfDNA | 1.95 | 0.182 | <0.001 | 1.3639–2.7800 |
| Recipient age (years) | 0.97 | 0.015 | 0.058 | 0.9423–1.0010 |
| Male recipient sex | 0.34 | 0.691 | 0.114 | 0.0865–1.3000 |
| Time from transplantation to biopsy (log-transformed) | 0.90 | 0.117 | 0.374 | 0.7159–1.1340 |
| Baseline eGFR (mL/min/1.73 m²) | 1.01 | 0.015 | 0.673 | 0.9772–1.0360 |
| Baseline proteinuria (log-transformed) | 1.10 | 0.280 | 0.738 | 0.6348–1.8990 |

## Supplementary Table S10. Outcome B – Proportional hazards test (no histology)

| **Variable** | **PH test p-value** |
| --- | --- |
| Time-dependent dd-cfDNA | 0.79 |
| Recipient age (years) | 0.38 |
| Sex | 0.28 |
| Time from transplantation to biopsy (log-transformed) | 0.51 |
| Baseline eGFR (mL/min/1.73 m²) | 0.25 |
| Baseline proteinuria (log-transformed) | 0.2 |
| GLOBAL | 0.29 |

**Model summary:** C-index=0.804 (SE 0.061); LR p=0.20; Wald p=0.001; Score p=0.05; Robust score p=0.40; Global PH p=0.16.

## Supplementary Table S11. Outcome B – Sensitivity analysis (chronicity domain only)

| **Variable** | **HR** | **Robust SE** | **p-value** | **95% CI** |
| --- | --- | --- | --- | --- |
| Time-dependent dd-cfDNA | 1.97 | 0.173 | 8.12e-05 | 1.4075–2.7680 |
| Recipient age (years) | 0.98 | 0.018 | 0.152 | 0.9418–1.0090 |
| Male recipient sex | 0.36 | 0.711 | 0.155 | 0.0903–1.4650 |
| Time from transplantation to biopsy (log-transformed) | 0.98 | 0.176 | 0.904 | 0.6938–1.3820 |
| Baseline eGFR (mL/min/1.73 m²) | 1.00 | 0.016 | 0.853 | 0.9714–1.0360 |
| Baseline proteinuria (log-transformed) | 1.18 | 0.320 | 0.612 | 0.6282–2.2040 |
| Chronicity (ci+ct+cv+cg) | 0.86 | 0.223 | 0.495 | 0.5552–1.3290 |

## Supplementary Table S12. Outcome B – Proportional hazards test (chronicity only)

| **Variable** | **PH test p-value** |
| --- | --- |
| Time-dependent dd-cfDNA | 0.74 |
| Recipient age (years) | 0.35 |
| Sex | 0.28 |
| Time from transplantation to biopsy (log-transformed) | 0.5 |
| Baseline eGFR (mL/min/1.73 m²) | 0.21 |
| Baseline proteinuria (log-transformed) | 0.15 |
| Chronicity (ci+ct+cv+cg) | 0.58 |
| GLOBAL | 0.16 |

# Outcome C: Graft failure

**Model summary:** C-index=0.853 (SE 0.057); LR p=0.002; Wald p=5e-14; Score p=1e-10; Robust score p=0.30; Global PH p=0.60.

## Supplementary Table S13. Outcome C – Primary model

| **Variable** | **HR** | **Robust SE** | **p-value** | **95% CI** |
| --- | --- | --- | --- | --- |
| Time-dependent dd-cfDNA | 3.42 | 0.275 | 7.53e-06 | 1.9964–5.8560 |
| Recipient age (years) | 1.05 | 0.046 | 0.307 | 0.9574–1.1480 |
| Male recipient sex | 2.79 | 1.142 | 0.368 | 0.2982–26.1810 |
| Time from transplantation to biopsy (log-transformed) | 1.47 | 0.255 | 0.133 | 0.8897–2.4190 |
| Baseline eGFR (mL/min/1.73 m²) | 1.00 | 0.031 | 0.936 | 0.9443–1.0640 |
| Baseline proteinuria (log-transformed) | 1.48 | 0.415 | 0.345 | 0.6564–3.3370 |
| TCMR/TI (t+i+v) | 0.99 | 0.146 | 0.941 | 0.7428–1.3170 |
| AMR/MVI (g+ptc+c4d) | 1.08 | 0.249 | 0.75 | 0.6640–1.7650 |
| Chronicity (ci+ct+cv+cg) | 0.87 | 0.200 | 0.499 | 0.5900–1.2930 |

## Supplementary Table S14. Outcome C – Proportional hazards test (primary model)

| **Variable** | **PH test p-value** |
| --- | --- |
| Time-dependent dd-cfDNA | 0.17 |
| Recipient age (years) | 0.95 |
| Sex | 0.25 |
| Time from transplantation to biopsy (log-transformed) | 0.25 |
| Baseline eGFR (mL/min/1.73 m²) | 0.14 |
| Baseline proteinuria (log-transformed) | 0.87 |
| TCMR/TI (t+i+v) | 0.96 |
| AMR/MVI (g+ptc+c4d) | 0.94 |
| Chronicity (ci+ct+cv+cg) | 0.81 |
| GLOBAL | 0.6 |

**Model summary:** C-index=0.861 (SE 0.052); LR p=2e-04; Wald p=3e-11; Score p=2e-11; Robust score p=0.10; Global PH p=0.60.

## Supplementary Table S15. Outcome C – Sensitivity analysis (no histology)

| **Variable** | **HR** | **Robust SE** | **p-value** | **95% CI** |
| --- | --- | --- | --- | --- |
| Time-dependent dd-cfDNA | 3.26 | 0.234 | 4.57e-07 | 2.0599–5.1620 |
| Recipient age (years) | 1.04 | 0.040 | 0.294 | 0.9642–1.1280 |
| Male recipient sex | 2.77 | 1.115 | 0.361 | 0.3112–24.6360 |
| Time from transplantation to biopsy (log-transformed) | 1.32 | 0.196 | 0.155 | 0.9000–1.9430 |
| Baseline eGFR (mL/min/1.73 m²) | 1.01 | 0.026 | 0.815 | 0.9560–1.0590 |
| Baseline proteinuria (log-transformed) | 1.40 | 0.424 | 0.428 | 0.6098–3.2070 |

## Supplementary Table S16. Outcome C – Proportional hazards test (no histology)

| **Variable** | **PH test p-value** |
| --- | --- |
| Time-dependent dd-cfDNA | 0.22 |
| Recipient age (years) | 0.95 |
| Sex | 0.28 |
| Time from transplantation to biopsy (log-transformed) | 0.24 |
| Baseline eGFR (mL/min/1.73 m²) | 0.17 |
| Baseline proteinuria (log-transformed) | 0.81 |
| GLOBAL | 0.6 |

**Model summary:** C-index=0.853 (SE 0.057); LR p=4e-04; Wald p=6e-14; Score p=5e-11; Robust score p=0.20; Global PH p=0.63.

## Supplementary Table S17. Outcome C – Sensitivity analysis (chronicity domain only)

| **Variable** | **HR** | **Robust SE** | **p-value** | **95% CI** |
| --- | --- | --- | --- | --- |
| Time-dependent dd-cfDNA | 3.41 | 0.275 | 8.36e-06 | 1.9875–5.8450 |
| Recipient age (years) | 1.05 | 0.047 | 0.319 | 0.9555–1.1500 |
| Male recipient sex | 2.93 | 1.115 | 0.336 | 0.3289–26.0210 |
| Time from transplantation to biopsy (log-transformed) | 1.42 | 0.270 | 0.19 | 0.8392–2.4140 |
| Baseline eGFR (mL/min/1.73 m²) | 1.00 | 0.030 | 0.94 | 0.9456–1.0620 |
| Baseline proteinuria (log-transformed) | 1.50 | 0.417 | 0.334 | 0.6606–3.3910 |
| Chronicity (ci+ct+cv+cg) | 0.89 | 0.200 | 0.579 | 0.6044–1.3250 |

## Supplementary Table S18. Outcome C – Proportional hazards test (chronicity only)

| **Variable** | **PH test p-value** |
| --- | --- |
| Time-dependent dd-cfDNA | 0.2 |
| Recipient age (years) | 0.94 |
| Sex | 0.26 |
| Time from transplantation to biopsy (log-transformed) | 0.26 |
| Baseline eGFR (mL/min/1.73 m²) | 0.14 |
| Baseline proteinuria (log-transformed) | 0.89 |
| Chronicity (ci+ct+cv+cg) | 0.79 |
| GLOBAL | 0.63 |
